# Effects of thermal environment on sleep and circadian rhythm

**DOI:** 10.1186/1880-6805-31-14

**Published:** 2012-05-31

**Authors:** Kazue Okamoto-Mizuno, Koh Mizuno

**Affiliations:** 1 Kansei Fukushi Research Center, Tohoku Fukushi University, 1-149-6 Kunimigaoka Aoba Sendai, Miyagi, 981-0935, Japan; 2 Department of Early Childhood and Primary Education, Tohoku Fukushi University, 6-149-1 Kunimi, Aoba-ku, Sendai, Miyagi, 989-3201, Japan

**Keywords:** Cold, Heat, Sleep, Thermal environment, Thermoregulation

## Abstract

The thermal environment is one of the most important factors that can affect human sleep. The stereotypical effects of heat or cold exposure are increased wakefulness and decreased rapid eye movement sleep and slow wave sleep. These effects of the thermal environment on sleep stages are strongly linked to thermoregulation, which affects the mechanism regulating sleep. The effects on sleep stages also differ depending on the use of bedding and/or clothing. In semi-nude subjects, sleep stages are more affected by cold exposure than heat exposure. In real-life situations where bedding and clothing are used, heat exposure increases wakefulness and decreases slow wave sleep and rapid eye movement sleep. Humid heat exposure further increases thermal load during sleep and affects sleep stages and thermoregulation. On the other hand, cold exposure does not affect sleep stages, though the use of beddings and clothing during sleep is critical in supporting thermoregulation and sleep in cold exposure. However, cold exposure affects cardiac autonomic response during sleep without affecting sleep stages and subjective sensations. These results indicate that the impact of cold exposure may be greater than that of heat exposure in real-life situations; thus, further studies are warranted that consider the effect of cold exposure on sleep and other physiological parameters.

## Review

The thermal environment is a key determinant of sleep because thermoregulation is strongly linked to the mechanism regulating sleep [1]. Excessively high or low ambient temperature (Ta) may affect sleep even in healthy humans without insomnia. Furthermore, disturbed nocturnal sleep affects not only daytime activities, but is also related to various adverse health effects, such as obesity [2], quality of life, and even mortality [3,4]. These findings indicate that maintaining a comfortable thermal sleep environment is important for sleep maintenance as well as daytime activities and health status. Among the various thermal environmental factors, the relationship between Ta and the use of clothing and beddings differs greatly between humans and animals. The effects of Ta on sleep stages differ depending on whether subjects are semi-nude or use bedding and clothing, making it difficult to extrapolate the results of animal studies related to thermal environment and sleep to humans. The use of clothing and beddings greatly aid in maintaining the body temperature at an acceptable thermal state in a variety of environments by providing thermal resistance for the human body from its environment [5]. In this review, based on our studies related to thermoregulation and sleep in humans, the effects of the thermal environment on sleep and circadian rhythm are discussed. The effects of heat and cold exposure, including bedding and clothing conditions along with their effects in the elderly, are of special interest, because these are the important factors that most impact sleep and thermoregulation.

### Sleep and thermoregulation

Many previous studies in humans indicate that sleep is strongly linked to thermoregulation (For examples, see [1,6]), which is primarily controlled by circadian rhythm and sleep regulation. Humans have a sleep-wake rhythm that is repeated in a 24-hour cycle. The core body temperature (Tcore), which also cycles along with the sleep-wake rhythm, decreases during the nocturnal sleep phase and increases during the wake phase repeatedly in a 24-hour circadian rhythm. Sleep is most likely to occur when Tcore decreases, while it hardly occurs during the increasing phases [7]. This relationship between the sleep-wake rhythm and the circadian rhythm of Tcore is important for maintaining sleep. At the normal sleep onset period in humans, Tcore decreases due to an underlying circadian rhythm, and sleep further induces this effect [8]. The driving force behind this Tcore decrease is the peripheral skin temperature (Tsk), which is rich in arteriovenous anastomoses and plays a central role in thermoregulation by adjusting blood flow to the skin [9]. Increased peripheral Tsk is largely due to reduced activation of noradrenergic vasoconstrictor tone, allowing greater inflow of heated blood from the core, thus facilitating heat loss to the environment through the skin surface [10]. The selective vasodilation of distal skin regions promotes the rapid onset of sleep [9] and is strongly associated with melatonin secretion [11]. Indeed, foot Tsk warming has been shown to reduce sleep onset latency [12], indicating that normal sleep onset is accomplished by increased peripheral heat loss and/or Tcore decrease [13]. The Tcore decrease in the sleep onset period is also strongly associated with cardiac autonomic activity. It has been suggested that changes in the cardiac autonomic nervous system precede sleep onset, which is strongly associated with changes in body temperature [14].

After sleep onset, Tcore gradually decreases further [8], while distal and proximal Tsk remain high [15]. The importance of maintaining Tsk at physiological range for sleep maintenance has been suggested [6]. In the elderly, only chest Tsk shows a significant decrease during sleep compared with the young [16,17], and slow wave sleep (SWS) decreases and wakefulness increases compared with the young, with increased insomnia [18]. The sleep parameters measured by actigraphy correlate only with chest Tsk, and decreased chest Tsk is associated with a decrease in the sleep efficiency index [19]. Interestingly, even a slight increase in proximal Tsk increases the amount of SWS and decreases the early-morning awakening in the elderly [20]. Sleep-related areas in the brain are associated with an increased Tsk within the physiological range during sleep [21]. Moreover, Tsk might act as an input signal to sleep-regulating systems [6]. These results indicate that slightly increasing the proximal Tsk may help alleviate sleep problems, especially in the elderly. The temperature and humidity of the microclimate between humans and bed covers (bed climate) also play crucial roles in creating a warm bed climate temperature to support increased Tsk and sleep [6]. The bed climate temperature and relative humidity are generally maintained around 32°C to 34°C, 40% to 60% relative humidity when normal sleep is obtained [22,23], which is in agreement with the comfort bed climate range suggested by Yanase [24].

Another important aspect to consider is that the thermoregulatory response during sleep differs depending on sleep stages. In animal studies, the thermoregulatory system is abolished during rapid eye movement sleep (REM) [25], due to a loss of thermosensitivity in the majority of the hypothalamic preoptic neurons [26]. In humans, thermosensitivity during REM is not completely depressed; however, sensitivity to hot or cold stimulation is reduced in REM compared to non-REM and wakefulness [27,28]. In addition, the sweat rate increases during SWS compared to other sleep stages [29], while delayed onset of sweating [30] and a decreased sweat rate [31] decrease evaporative heat dissipation and reduce heat tolerance during REM. Interestingly, the decreased sweat rate during REM is observed prior to REM stage onset [32]. Considering that skin sympathetic nerve activity (SSNA) contains sudomotor activity synchronous with vasodilator activity [33], this result indicates that SSNA might precede the sleep stage shift corresponding with heart rate variability (HRV) preceding the sleep stage shift at the sleep onset period [14]. Changes in the sensitivity of the sweat response depending on sleep stages are considered to be a central drive effect, since no peripheral change in sweat gland levels has been observed [34]. In cold exposure, shivering during sleep is confined to stages 1 and 2 and is not observed in SWS and REM [35], while the Tsk of the extremities is decreased during REM compared to that at control conditions [36]. These results indicate that REM and thermoregulation are mutually exclusive and partly explain the decrease in REM observed during heat or cold exposure, bearing in mind that REM is more sensitive to Ta than other sleep stages.

Although physiological thermoregulation related to sleep has been well defined, behavioral thermoregulation during sleep remains unclear. Limited behavioral thermoregulation reduces the thermoregulatory response during sleep compared to wakefulness. However, Ta increases or decreases during sleep significantly decrease or increase, respectively, the areas of the body covered by bed covers, with the neck, shoulder and upper extremities showing higher sensitivity than lower extremities and the trunk [37]. In heat exposure, lateral body position increases compared to the supine position, possibly because this position may decrease the contact area between the body and the mattress [38]. These results suggest that behavioral thermoregulation is active during sleep and that bed cover behaviors and body position may have an important role. Considering that poor sleepers spend more time on their backs with their heads straight, sleep positions may be related to sleep quality [39]. It is interesting to consider these behaviors in various thermal environments during sleep, since it may be an important sleep variable that would aid in our further understanding of thermoregulation during sleep in humans.

### Effects of heat exposure

Increases in wakefulness are greater in cold Ta than in heat , suggesting that the impact of cold exposure is greater than that of heat exposure. Ta higher or lower than the thermal neutral temperature (29°C) have been shown to increase wakefulness and decrease REM and SWS in semi-nude subjects [40]. However, these results are based on semi-nude subjects and exclude the effects of bed covers and clothing. In real-life situations where bed covers and clothing are used, sleep is actually disturbed during heat exposure rather than cold exposure in the young [41], as well as in the elderly [19]. The increased wakefulness and decreased SWS and REM are stereotypical effects that are observed in heat exposure [42]. These effects on sleep stages are concentrated in the initial segment rather than the later segment of sleep. One possible explanation for this is that sleep disruption in the initial sleep segment leads to an increased demand, which may overcome the thermal stress in the later segment of sleep [42,43]. Heat-related sleep disruptions do not adapt even after 5 days of continuous daytime and nocturnal heat exposure [44]. Furthermore, the effect on SWS does not change after partial sleep deprivation where sleep pressure is increased [45]. These results suggest a strong effect of heat load on sleep stages, which is related to thermoregulation during sleep. Thus, wakefulness is the only stage that can cope with an increased thermal load [26] and that wakefulness replaces SWS and REM to maintain homeothermy.

Heat load suppresses the decrease in Tcore and increases Tsk and whole body sweat loss during sleep [43,46]. Although Tsk increases at the onset of sleep, high Ta suppresses heat loss to the environment through the skin surface, thereby suppressing the decrease in Tcore. This suppressed decrease in Tcore may disturb sleep at the initial segment of sleep. The increased Tsk is largely due to increased skin blood flow, which is regulated primarily through two pathways in the sympathetic nervous system: the noradrenergic vasoconstrictor system and the active vasodilator system. Increased Tsk during sleep in heat exposure may be largely due to an increased active vasodilator system. In subjects that are awake, an active vasodilator system is responsible for most of the vasodilatory responses to heat stress [47]. The SSNA contains vasodilator activity synchronous with sudomotor activity [33] and may lead to an increase in whole body sweat loss. Taken together, these results suggest the possibility that increased vasodilator activity may be related to increased Tsk and wakefulness due to heat exposure during sleep. Because sleep distribution is controlled by both peripheral and central driving effects [13,48], it is possible that increased active vasodilation and Tcore both increase wakefulness during heat exposure. These results support the notion that, although sleep states affect thermoregulation, thermoregulation equally affects the mechanism governing sleep [1].

One of the most important factors that increase heat stress during sleep is the humidity. Humid heat exposure further increases wakefulness, decreases REM and SWS, and excessively suppresses the decrease in Tcore, whereas Tsk and whole body sweat loss are not affected [43]. Humid heat exposure most probably increases heat stress because of the difference in the sweat response caused by the humidity. Decreased ambient humidity allows sweat to evaporate, thereby dissipating the heat, whereas increased humidity does not allow the sweat to evaporate, causing the skin to remain wet. The dripping sweat and increased skin wetness decrease the sweat response due to hidromeiosis preventing dehydration. These results indicate the importance of taking humidity into account, especially in Japan and many other Asian countries that experience humid heat in the summer.

**Figure 1:**
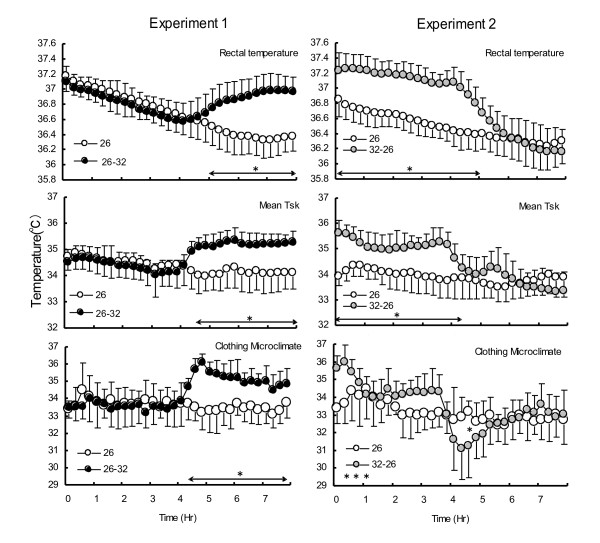
**Effects of humid heat exposure at different segment of sleep on thermoregulation.** Experiments 1 (26°C 60%RH stable (26), compared with 26°C 60% in the initial segment and 32°C 80%RH in the later segment of sleep (26 → 32)) and experiments 2 (26°C60%RH stable (26), compared with 32°C 80%RH in the initial segment and 26°C60%RH in the later segment of sleep (32 → 26)).. Reprinted from Okamoto-Mizuno K, Tsuzuki K, MizunoK, Iwaki T: Effects of partial humid heat exposure during different segments of sleep on human sleep stages and body temperature. Physiol Behav 2005, 83:759–765 with kind permission from Elsevier.

Interestingly, although the exposure time is the same, the effect of humid heat is greater in the initial segment than in the later segment of sleep. Humid heat exposure during the later segment of sleep increases wakefulness in that segment [49,50]. In contrast, humid heat exposure in the initial segment decreases SWS in that segment and increases wakefulness in both segments [50]. Furthermore, decreased SWS in the initial segment of sleep tends to increase it in the later segment of sleep. Thus, humid heat exposure in the initial segment of sleep appears to change the polarity of SWS [50]. These effects on sleep stages can be explained by thermoregulation and microclimate temperature (temperature and humidity of the microclimate between skin and the clothing) (Figure 1). Humid heat exposure in the later segment causes a decrease in Tcore in the initial segment, followed by an increase in Tcore, Tsk and microclimate temperature in the later segment. Humid heat exposure in the initial segment suppresses the decrease in Tcore, whereas Tsk and the microclimate temperature increase lead to a decrease in SWS and an increase in wakefulness. Furthermore, the sharp decrease in Tcore, Tsk and the microclimate temperature in the later segment of sleep may also increase wakefulness. The chilling effects of a decrease in Ta and humidity and a reduction in clothing insulation due to wetness [51] caused by sweating at initial humid heat exposure may also be a possibility for the increase in wakefulness. Another possible contributing factor for decreased Tsk and Tcore in the later segment of sleep could be that a decrease in Ta coincides with a time when REM sleep generally increases, since Tsk decreases are greater in REM compared to other sleep stages [36]. In Japan, a majority use air conditioning only for a few hours after going to sleep [52] due to the notion that being exposed to air conditioning is unhealthy [53], and 90% of Japanese people are interested in saving energy [54]. These results indicate that if air conditioning use is limited, then it should be used during the initial segment of sleep. Furthermore, when air conditioning is used in the later segment of sleep, drying off the sweat and changing clothing are essential to avoid chilling effects.

In the elderly, even mild heat exposure increases wakefulness and decreases REM [55]. Since the elderly exhibit a decreased amount of SWS even under normal conditions, SWS is not thought to be affected by heat exposure. Sleep consolidation decreases in older men [56], which in turn increases susceptibility to external arousal stimuli during sleep [57]. These changes in sleep may decrease the thermal awakening threshold. Another reason for this could be reduced heat tolerance in older men, since most heat exposure studies have found a reduced ability to regulate Tcore in subjects who are awake (for example, see [58]). Mild heat exposure further suppresses the decline in Tcore, increases Tsk, and increases whole body sweat loss by two-fold in the elderly [55]. The increase in nocturnal Tcore [59] and the attenuation of the nocturnal drop in Tcore [60] may underlie age-related declines in sleep maintenance and sleep quality. Furthermore, the period of overnight fasting results in mild dehydration in older men even under normal conditions [61], suggesting that increased whole body sweat loss during sleep in mild heat exposure may be related to dehydration in the elderly. Although further study to directly compare older and younger men is needed, it appears quite likely that sleep in older men is more affected by heat exposure than in younger men. These results indicate that the Ta during sleep warrants particularly careful consideration in older men, especially since decreased sleep duration in the older men is related to reduced quality of life and mortality [3,4]. Ta for the elderly should also take into account clothing conditions; elderly Japanese people wear more than two layers of underwear under nightwear even in summer [62].

### Effects of cold exposure

The difference between cold exposure and heat exposure is that cold exposure mainly affects the later segment of sleep, where REM is dominant [63]. In semi-nude subjects, cold exposure mainly affects REM due to suppression of the thermoregulatory response [35,40]. SWS is not affected because it predominates in the initial segment of sleep [64]. In thermoregulation during sleep, Tcore decreases through the night [36] as the Ta decreases [65]. However, in real-life situations people generally use clothing and bed covers during sleep in cold exposure. In studies using clothing and/or bedding, no significant difference was observed in sleep in a Ta range of 13°C to 23°C [66] and 3°C to 17°C [67]. Also, no significant difference in sleep quality measured by actigraphy was observed between 9°C and 20°C in the elderly [19]. These results indicate that, in real-life situations, cold exposure does not affect sleep. This is related to the fact that despite large changes in Ta the bed climate temperature remained relatively constant [66]. The use of bed covers allows for the development of an isolated high bed climate temperature, which is critical for maintaining sleep [6] as well as determining sleep quality [68].

**Figure 2:**
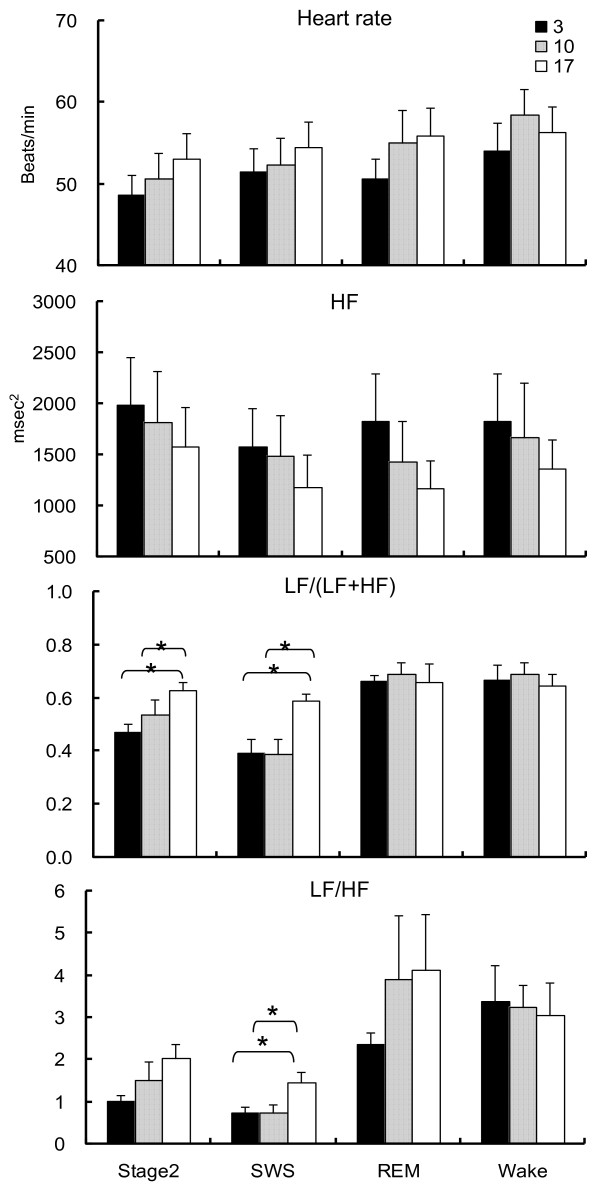
**Changes of heart rate and high frequency, percentage of low frequency and ratio of low frequency to high frequency components in three conditions.** The vertical line represents the standard error. *indicates the significant level after Friedman test; *P* <0.05. ^†^indicates the significant level after Scheffe’s post-hoc test; *P* <0.05. Reprinted from Okamoto-Mizuno K, Tsuzuki K, Mizuno K, Ohshiro Y: Effects of low ambient temperature on heart rate variability during sleep in humans. Eur J Appl Physiol 2009, 105:191–197 with kind permission from Springer Science and Business Media.

However, our study indicates that cold exposure significantly changes cardiac autonomic activity during sleep, without affecting the sleep stages (Figure 2) [67]. With regard to cardiac autonomic activity based on the HRV index, the ratio of the low frequency (LF) to high frequency (HF) band (LF/HF) significantly decreases during stage 2 and SWS, while the percentage of the LF component (LF/(LF + HF)) significantly decreases during SWS as the Ta decreases from 17°C to 3°C. In contrast, no significant effect is observed during REM or wakefulness. These results may indicate that cardiac parasympathetic activity predominates under cold exposure during stage 2 and SWS, although the results of the LF/HF and LF/(LF + HF) should be interpreted with caution. The dominant parasympathetic activity during stage 2 and SWS may be related to at least three factors: cold stimulation of the head since sufficient thermal insulation of the body is obtained from bedding and clothing; cold air inhalation; and whole body cooling. First, cold stimulation of the face, a unique reflex referred to as cold face test, increases the cardiac parasympathetic activity and the peripheral skin SSNA simultaneously and integrates the trigeminal brain stem reflex arc in wakeful subjects [69]. Additionally, this reflex activates reflex centers located in the medullary region and induces bradycardia [70-72]. Furthermore, the concomitant increase in the SSNA leads to vasoconstriction, an increase in blood pressure [70-72] and a significant increase in the HF component [69,73]. Second, inhalation of cold air may increase muscle sympathetic nervous activity and blood pressure in wakeful subjects [74]. Third, whole body cooling may also be related to increases in systolic and diastolic blood pressure and decreases in heart rate in wakeful subjects [75]. Considering that approximately 70% of sleep time comprises stage 2 and SWS, the cardiac parasympathetic activity may be dominant during sleep in cold exposure.

These findings could partly explain the adverse cardiac events that peak during the colder periods in the winter season [76,77]. Mortality due to ischemic heart disease is not related to low outside temperature, but to low living-room temperatures and limited bedroom heating in the winter [78]. Cold climates increase blood pressure [75] as well as the levels of hematological factors that favor arterial thrombosis [79] and fibrinogen synthesis [80]. It has been suggested that the onset of major cardiovascular events is triggered by sleep stage-dependent fluctuations in the autonomic nervous system [81]. Significantly dominant cardiac parasympathetic activity during stage 2 and SWS, which is not observed during REM and wakefulness in cold exposure, suggests marked variations in HRV during the transition from stage 2 to REM and wakefulness. The marked rise in HRV during transition from non-REM to awakening and REM favors adverse cardiac events [82]. Further study considering the relationship between the increased incidence of adverse cardiac events and marked variations in HRV during transition from non-REM to wakefulness and REM may provide insights into understanding the increase in cardiac events peaking in winter. Besides HRV, the lack of nocturnal decline in blood pressure is also related to an increase in end-organ damage and cardiovascular events [83,84]. Considering that the cold face test, inhaling cold air and whole body cooling accompany increased blood pressure, it would be interesting to investigate whether cold exposure suppresses blood pressure decline during sleep. It is extremely important to note that Ta in winter should be maintained at a level higher than 10°C. However, the most difficult aspect of cold exposure is that sleep is not disturbed. The impact on the cardiovascular response may be occurring without subjective sensation, suggesting that cold exposure may have more impact than heat exposure. In Japan, bedroom Ta falls to as low as 3°C in the suburbs [53]. Excessive layers of underwear under night wear and bed covers are common observations in elderly Japanese people [85] and this behavior supports sleep in cold exposure. Indeed, about 50% of elderly Japanese people use two to six layers of underwear and three to five layers of bed covers [85]. The most important aspect of this is whether HRV during sleep in elderly people habitually sleeping in cold Ta is affected by cold exposure or is somewhat adapted to it. Further study on measuring HRV in the elderly habitually sleeping in cold Ta at home may thus be required.

### Ambient temperature and circadian rhythm

The environmental light-dark cycle is the principal environmental synchronizer of the circadian pacemaker in humans as well as other species [7]; however, the effects of social cues and other nonphotic entrainment, including Ta, on human circadian system are less understood. Twenty-four hours of warm Ta increases activity, suggesting that Ta has a masking effect on circadian activity rhythms in animal studies [86]. In high and cool Ta cycles, Ta acts as a weak synchronizer in laboratory rats [87] and mice [88]. These results suggest that, at the least, Ta may have masking effects on circadian activity rhythms in homeothermic animals. In humans, many studies indicate that different Ta cycles during sleep within the thermoneutral Ta range can affect Tcore. Decreased Ta occurring a few hours before and after sleep onset and increased Ta occurring around wake-up time increases the decline in Tcore and advances the Tcore nadir compared to constant Ta [89,90] and/or the opposite Ta cycle [91,92]. The effects of these cyclic Ta changes do not significantly affect sleep stages [89], or increase SWS compared to constant Ta [90]. These results indicate that cyclic Ta changes do not induce any adverse effects on sleep stages at least within the thermoneutral temperature range. Interestingly, studies by Dewasmes *et al*. [89,93] showed that cyclic Ta, as opposed to constant Ta, advanced the minimum Tcore by 143 minutes and the propensity for REM. The REM cycle length changes depending on Ta with a delayed REM cycle in low Ta compared with high Ta [94], and the REM propensity has a close relationship with body temperature rhythm [95]. These results indicate that Ta itself as well as cyclic Ta change may advance circadian Tcore and/or REM and that the thermoregulatory system may have effects on phase advancing mechanisms.

One possible explanation for these effects of cyclic Ta change on Tcore may be the reduced thermoregulation during sleep compared to wakefulness [28]. No significant difference between sleep stages at Ta of 13°C to 23°C is observed, although Tcore clearly decreases as the Ta decreases [66]. Not only Ta but also bed mattress properties with decreased thermal insulation result in significantly decreased Tcore without affecting sleep stages [96]. Although sleep stages were not measured, effects of different types of quilts [97] and clothing [98] during sleep decreased Tcore under decreased thermal insulation conditions. The effects of heat exposure during sleep are also greater compared to the waking state with an increase of a few degrees in Ta above the thermal neutral zone affecting Tcore during sleep [31]. These results indicate that Tcore during sleep may be sensitive to Ta change as well as clothing and bedding thermal insulation. It is important to note that these effects on Tcore do not always affect sleep stages in parallel. During cyclic Ta changes, Tcore may be affected by peripheral skin blood flow that regulates the circadian rhythm of Tcore [99]. Increased and decreased dry heat loss from changes in the peripheral Tsk has been suggested as one possible effect of cyclic Ta change on Tcore during sleep [100]. However, further precise study on peripheral Tsk under cyclic Ta changes together with sleep stages, especially with REM propensity and cycle, melatonin secretion and bed climate, is needed.

Interestingly, it has been suggested that time memory for heat exposure exists in the human thermoregulatory system, and that autonomic thermoregulation in Tcore changes during the previous heat exposure period without actual temperature stimuli [101]. It would be interesting to know whether continuous phase shifting effect of cyclic Ta changes during sleep may keep time memory and whether this phase shifting effect continues even under constant Ta. Furthermore, effects of cyclic Ta change on Tcore are limited during sleep, and further study is warranted to determine its effects on the wakeful state. Results from blind individuals indicate that, although nonphotic stimuli can exert a small, but significant resetting response, these effects are weaker than light stimuli in affecting the human circadian pacemaker [102]. Indeed, cyclic Ta change combined with a gradual light-dark intensity cycle indicates a stronger effect of light compared with Ta [103]. In real-life situations, Ta and light change in a 24-hour cycle may involve the circadian system in humans. It has been suggested that in, addition to light, the daily rise and fall in environmental temperature could be an essential input to the circadian clock [104]. Since thermoregulatory mechanisms are strongly related with the circadian timing system, the Ta change may be an essential entrainment input additional to light environment, or at least exert masking effects on the circadian system.

## Conclusions

Heat exposure affects SWS and REM, whereas cold exposure does not affect sleep stages. Considering that a Ta of 32°C with 80% relative humidity affects only SWS without affecting REM [46], heat affects SWS first, whereas REM may be well-preserved in real-life situations. Sleep disturbance during heat exposure may lead to behavioral thermoregulation in humans, for example, using an air conditioner to decrease Ta. However, during cold exposure, the cardiac autonomic response may be affected without affecting sleep stages and subjective sensations, and so not trigger behavioral thermoregulation to control Ta. This indicates that the impact of cold exposure may be greater than that of heat; thus, further studies are warranted to consider the effect of cold exposure on sleep and other physiological parameters.

## Competing interests

The authors declare that they have no competing interests.

## Authors’ contributions

KO-M has made substantial contributions to the manuscript. KM has been involved in drafting the manuscript and revising it critically for important intellectual content. Both authors read and approved the final manuscript.
